# Knowledge, attitudes, and practices among the general community population toward heatstroke

**DOI:** 10.3389/fpubh.2024.1373025

**Published:** 2024-05-24

**Authors:** Yangfeng Xu, Jianping Chen, Jinkang Du, YunYing Jin

**Affiliations:** ^1^Department of Emergency, Dongyang People's Hospital, Dongyang, China; ^2^Intensive Care Unit, Dongyang People's Hospital, Dongyang, China

**Keywords:** knowledge, attitudes, practices, heatstroke, cross-sectional study

## Abstract

**Background and objective:**

Heatstroke (HS) is a life-threatening condition resulting from thermal injury within the body, and it is associated with a significantly high mortality rate. This study aimed to assess the knowledge, attitudes and practices (KAP) among the general community population toward heatstroke.

**Methods:**

The web-based cross-sectional study was conducted between September 2023 and October 2023 at the Emergency Department of Dongyang People's Hospital. A self-designed questionnaire was developed to collect demographic information of the general community population and to assess their knowledge, attitudes and practices toward heatstroke.

**Results:**

A total of 1,356 valid questionnaires were collected. Among the participants, 875 (64.53%) were female, and 496 (36.58%) had regular exercise. The mean knowledge, attitudes and practices scores were 12.73 ± 1.42 (possible range: 0–14), 33.74 ± 2.91 (possible range: 8–40) and 34.65 ± 5.30 (possible range: 8–40), respectively. The structural equation model demonstrated that education had direct effects on knowledge (β = 0.017, *p* < 0.001), attitudes (β = 0.123, *p* < 0.001), and practices (β = −0.094, *p* < 0.001). Moreover, knowledge had direct effects on attitudes (β = 1.920, *p* < 0.001), and attitudes had direct effects on practices (β = 0.642, *p* < 0.001).

**Conclusion:**

The findings revealed that the general community population have sufficient knowledge, active attitudes and proactive practices toward the heatstroke. However, there is still room for improvement and it is necessary to develop and implement educational initiatives and interventions designed to further enhance their KAP toward heatstroke.

## Introduction

Heatstroke (HS) arises when the human body's temperature regulation system fails due to excessive environmental heat exposure. This failure leads to a dangerous elevation in core body temperature (Tcore), surpassing 40.6°C, which in turn can cause central nervous system dysfunction, manifesting as delirium, convulsions, and coma. As a severe condition resulting from internal thermal injury, heatstroke presents a significant life-threatening risk and is associated with a notably high mortality rate (1, 2).

Exposure to a hot environment is identified as a direct precursor to heatstroke (3). Due to the progressive impact of global warming, the incidence of fatalities from heat waves is increasing. For instance, in August 2003, an extended and intense heat wave in Europe was associated with 14,800 heat-related deaths in France alone (4). Another study highlights the relationship between HS and heat waves, noting that the relative risk of HS during heat wave days, as opposed to non-heat wave days, significantly declined from 71.0 in 1999 to 3.5 in 2010 (5). In China, the epidemiology of heatstroke exhibits distinctive patterns due to the country's vast geographical diversity and climatic variations. High daily maximum temperatures are closely linked to the incidence of heatstroke, with a 30.5% excess risk for each 1°C increment over the heatwave threshold (6). Urban areas, such as Shaoxing, experience a predominance of mild heatstroke cases, although severe cases and fatalities are not uncommon (7). Heat-related illnesses in China are concentrated in urban regions, particularly around the Yangtze River, with heightened vulnerability observed in the older adult and males aged 45–64 (8). This heightened susceptibility can be attributed to the aging process of human tissues and organs, which leads to a diminished capacity for heat dissipation (9). Consequently, this reduction in thermoregulatory efficiency accelerates the increase in core body temperature during episodes of heat exposure.

Timely and effective intervention holds paramount significance in enhancing the survival rate and prognosis of heatstroke patients (4). Timely and appropriate intervention can mitigate mortality and enhance the prognosis of heatstroke patients. Initiating treatment for heatstroke at the earliest possible stage, particularly in prehospital settings such as the home, is crucial (10). Before the arrival of professional medical assistance, on-site responders are instrumental in providing immediate care to individuals suffering from heatstroke. Their roles include critical actions such as extricating patients from the hot environment and implementing cooling strategies, for instance, the use of ice blankets. Currently, there is a lack of research examining the knowledge base of the general population regarding heatstroke and the corresponding practical measures that should be taken in response.

Knowledge, Attitudes, and Practices (KAP) framework, which serves as a crucial tool in public health for assessing and enhancing the awareness, attitudes, and practices of a population regarding specific health issues, offering invaluable insights essential for designing targeted and effective health interventions (11, 12). Therefore, this study aimed to explore the KAP among the general community population toward heatstroke. This exploration sought to gauge the existing level of awareness and prevalent attitudes and practices, thereby providing valuable insights for the development of tailored educational and preventive strategies.

## Methods

### Study design and participants

This cross-sectional survey was conducted between September 2023 and October 2023 at the Emergency Department of Dongyang People's Hospital. The study was ethically approved by the Ethics Committee of Dongyang People's Hospital (Approval no. 2023-YX-289) and informed consent was obtained from the study participants.

The inclusion criteria for this study were as follows: (1) participants aged 18 years or above, (2) having access to the internet to complete the web-based questionnaire, and (3) volunteering for participation in the study and demonstrate an understanding of the questionnaire. Data collection was conducted anonymously. To avoid duplicate responses, an IP restriction was applied, meaning that the survey could be filled out only once from any given IP address.

### Questionnaire

The questionnaire was crafted following the guidelines outlined in the Expert consensus on the diagnosis and treatment of Heatstroke in China (13) and pertinent literature on heatstroke (14–16). The initial draft underwent refinement through feedback from two senior experts, all holding the title of associate professor, with expertise in emergency medicine. Following these revisions, a preliminary trial was conducted on a limited scale (*n* = 38), yielding a Cronbach's alpha coefficient value of 0.738, indicative of good internal consistency.

The final questionnaire, written in Chinese, encompassed four dimensions: demographic information, knowledge, attitudes and practices. The demographic section comprised 14 items, while the knowledge, attitudes, and practices dimensions included 16, 8, and 8 items, respectively. Notably, questions K2 and K14 were intentionally designed as trap questions, presenting precisely opposite meanings, both of them were excluded from scoring and subsequent statistical analyses. Participants who selected “right” or “wrong” for both questions were considered to have a logical conflict and were subsequently excluded from the survey. Consequently, the knowledge items were assigned 1 point for a correct answer and 0 points for incorrect responses, resulting in a possible score range of 0–14. The attitude items were scored on a five-point Likert scale ranging from very positive (5 points) to very negative (1 point), with a possible score range of 8 to 40. Similarly, the practice items were scored on a five-point Likert scale, ranging from very consistent (5 points) to very inconsistent (1 point), with a possible score range of 8 to 40. Sufficient knowledge, active attitudes, and proactive practices were defined as achieving scores surpassing 75% of the maximum possible score in each respective section (17).

The data were gathered through an online questionnaire administered via Sojump (http://www.sojump.com). This questionnaire was disseminated through WeChat platform.

### Statistical analysis

STATA 17.0 (Stata Corporation, College Station, TX, USA) was used for statistical analysis. The continuous variables were expressed as mean ± standard deviation (SD), and the categorical variables was expressed as *n* (%). The continuous variables conformed to a normal distribution were tested by the *t*-test or ANOVA. In multivariate analysis, 75% of the total score was used as the cut-off value. Pearson correlation was used to analyze the correlation between knowledge, attitudes, and practices. The structural equation model (SEM) for evaluating knowledge, attitudes, and practices regarding heatstroke among the general community population was established using AMOS 24.0 (IBM, NY, United States). The model fitting was evaluated with CMIN/DF (Chi-square fit statistics/degree of freedom), RMSEA (root mean square error of approximation), IFI (incremental fix index), TLI (Tucker-Lewis index) and CFI (comparative fix index). Two-sided *p* < 0.05 were considered statistically significant.

## Results

In this study, 1,356 questionnaires were collected. Among the participants, 875 (64.53%) of them were female and the average age was 39.73 ± 9.81 years old. A total of 849 (62.61%) presented BMI within the range of 18.5 to 23.9 kg/m^2^. A majority, comprising 699 individuals (51.55%), were indoor workers. Notably, 1,026 participants (75.66%) reported no habits of smoking or drinking. Regular physical activity was reported by 496 participants (36.58%), and 1,186 (87.46%) were not aware of any recent heatstroke incidents around them recently. The mean knowledge, attitude and practice scores were 12.73 ± 1.42 (possible range: 0–14), 33.74 ± 2.91 (possible range: 8–40) and 34.65 ± 5.30 (possible range: 8–40), respectively (Table 1).

**Table 1:** Knowledge, attitudes, and practices scores and demographic characteristics.

	**N (%)**	**Knowledge, mean ±SD**	***P***	**Attitude, mean ±SD**	***P***	**Practice, mean ±SD**	***P***
***N** **=*** **1, 356**							
**Total score**		12.73 ± 1.42		33.74 ± 2.91		34.65 ± 5.30	
**Gender**			0.892		0.306		0.059
Male	481 (35.47)	12.72 ± 1.36		33.63 ± 2.93		34.28 ± 5.71	
Female	875 (64.53)	12.73 ± 1.45		33.80 ± 2.90		34.85 ± 5.06	
**Age (years)**	39.73 ± 9.81						
**BMI (kg/m** ^**2**^ **)**			0.207		0.961		0.418
< 18.5	111 (8.19)	12.46 ± 1.62		33.82 ± 2.80		35.36 ± 4.81	
18.5–23.9	849 (62.61)	12.76 ± 1.39		33.71 ± 2.85		34.64 ± 5.33	
24–27.9	329 (24.26)	12.72 ± 1.32		33.77 ± 3.08		34.40 ± 5.48	
≥28	67 (4.94)	12.72 ± 1.77		33.85 ± 3.04		34.85 ± 4.86	
**Residence**			0.002		0.027		0.309
Rural	542 (39.97)	12.58 ± 1.47		33.53 ± 2.89		34.47 ± 5.50	
Urban	814 (60.03)	12.82 ± 1.37		33.88 ± 2.92		34.77 ± 5.16	
**Marital status**			0.524		0.160		0.068
Unmarried	146 (10.77)	12.77 ± 1.03		33.86 ± 3.03		33.75 ± 6.28	
Married	1166 (85.99)	12.73 ± 1.45		33.76 ± 2.89		34.78 ± 5.16	
Other	44 (3.24)	12.50 ± 1.70		32.93 ± 3.04		34.11 ± 5.18	
**Education**			< 0.001		< 0.001		0.571
Junior high school and below	138 (10.18)	12.22 ± 1.71		32.59 ± 3.02		34.43 ± 5.17	
High school/Technical school	234 (17.26)	12.35 ± 1.72		32.56 ± 2.93		34.29 ± 5.14	
College/Bachelor's degree	948 (69.91)	12.89 ± 1.26		34.14 ± 2.78		34.78 ± 5.35	
Master's degree and above	36 (2.65)	12.89 ± 1.01		35.36 ± 2.10		34.33 ± 5.62	
**Occupation**			< 0.001		< 0.001		0.092
Outdoor worker (e.g., sanitation worker, traffic police, construction worker, etc.)	247 (18.21)	12.42 ± 1.62		33.30 ± 3.05		35.25 ± 5.28	
Indoor worker (clerk, accountant, designer, etc.)	699 (51.55)	12.92 ± 1.25		34.17 ± 2.80		34.70 ± 5.42	
Occupation involving both indoor and outdoor activities	191 (14.09)	12.83 ± 1.26		33.69 ± 2.77		34.40 ± 5.01	
Unemployed, retired, or other non-working situations	219 (16.15)	12.35 ± 1.66		32.93 ± 2.97		34.05 ± 5.13	
**Monthly per capita income (CNY)**			< 0.001		< 0.001		0.023
< 2,000	71 (5.24)	11.92 ± 1.84		32.46 ± 3.29		32.66 ± 6.82	
2,000–5,000	369 (27.21)	12.71 ± 1.35		33.47 ± 2.93		34.69 ± 4.97	
5,000–10,000	533 (39.31)	12.80 ± 1.35		33.79 ± 2.83		34.68 ± 5.24	
10,000–20,000	249 (18.36)	12.78 ± 1.47		34.24 ± 2.78		34.99 ± 5.17	
>20,000	134 (9.88)	12.81 ± 1.38		34.06 ± 2.93		34.87 ± 5.61	
**Medical insurance**			< 0.001		0.005		< 0.001
Uninsured	59 (4.35)	11.86 ± 1.60		32.88 ± 3.25		32.88 ± 5.59	
Social insurance only	670 (49.41)	12.71 ± 1.49		33.60 ± 2.95		34.31 ± 5.45	
Comprehensive coverage	627 (46.24)	12.83 ± 1.28		33.97 ± 2.81		35.19 ± 5.05	
**Smoking or drinking**			0.392		0.630		0.220
Smoking only	84 (6.19)	12.77 ± 1.45		33.90 ± 2.73		34.88 ± 5.71	
Drinking only	113 (8.33)	12.59 ± 1.33		33.42 ± 3.06		33.75 ± 5.50	
Both	133 (9.81)	12.58 ± 1.51		33.77 ± 2.93		34.34 ± 5.65	
Neither	1026 (75.66)	12.76 ± 1.41		33.76 ± 2.91		34.77 ± 5.19	
**Regular exercise**			0.054		0.852		< 0.001
Yes	496 (36.58)	12.63 ± 1.44		33.72 ± 2.93		35.45 ± 4.98	
No	860 (63.42)	12.78 ± 1.40		33.75 ± 2.90		34.19 ± 5.43	
**Frequency of medical check-ups**			0.046		0.012		0.001
Every six months or less	801 (59.07)	12.79 ± 1.43		33.91 ± 2.86		35.06 ± 5.09	
Once a year	555 (40.93)	12.63 ± 1.40		33.50 ± 2.96		34.07 ± 5.55	
**Average nightly sleep duration in the past week**			0.005		0.016		0.276
5 h or less	102 (7.52)	12.36 ± 1.52		32.95 ± 3.09		34.00 ± 5.21	
5–7 h (including 7 h)	891 (65.71)	12.80 ± 1.39		33.83 ± 2.89		34.61 ± 5.33	
7 h or more	363 (26.77)	12.64 ± 1.44		33.75 ± 2.87		34.93 ± 5.26	
**Has there been any incidence of heatstroke?**			0.268		0.056		0.008
Personal experience of heatstroke has occurred.	51 (3.76)	12.76 ± 1.84		34.18 ± 2.63		35.49 ± 4.76	
Heatstroke has been experienced by family members or friends.	119 (8.78)	12.92 ± 1.25		34.27 ± 2.53		35.96 ± 3.93	
There have been no recent reports of heatstroke in the surrounding area.	1,186 (87.46)	12.70 ± 1.41		33.67 ± 2.95		34.48 ± 5.42	

The three knowledge items with the highest correctness rates were as follows: “On-site treatment for heatstroke patients should involve laying them flat, tilting their head to one side, unfastening buttons and belts, and removing outer clothing to facilitate breathing and heat dissipation.” (K12) with 98.16%, “Environments with high temperatures and high humidity are prone to induce heatstroke.” (K3) with 97.42%, and “Outdoor laborers (such as farmers, construction workers) or individuals engaging in high-intensity sports (such as athletes or trainees in military exercises) are more susceptible to heatstroke.” (K6) with 97.27%. The three items with the lowest correctness rates were “Once heatstroke occurs, even in extremely hot environments, the patient should not be moved.” (K11) with 71.02%, “If heatstroke-induced coma occurs during physical training, and the person quickly regains consciousness, hospital treatment may not be necessary.” (K15) with 75.22%, and “Inadequate rest time may induce heatstroke.” (K8) with 85.99% (Table 2).

**Table 2:** Knowledge.

	**Correctness rate N (%)**
K1. Common symptoms of heatstroke include skin burning sensation, impaired consciousness (such as delirium, convulsions, coma), and multi-organ dysfunction.	1,296 (95.58)
K3. Environments with high temperatures and high humidity are prone to induce heatstroke.	1,321 (97.42)
K4. Older individuals and those with weakened physical conditions, as well as children, are more susceptible to heatstroke.	1,273 (93.88)
K5. People with underlying health conditions (such as hyperthyroidism, hypohidrosis, severe skin diseases, etc.) are more prone to heatstroke.	1,292 (95.28)
K6. Outdoor laborers (such as farmers, construction workers) or individuals engaging in high-intensity sports (such as athletes or trainees in military exercises) are more susceptible to heatstroke.	1,319 (97.27)
K7. Taking certain medications that affect temperature regulation may be a risk factor for inducing heatstroke, so it is important to check the medication instructions.	1,278 (94.25)
K8. Inadequate rest time may induce heatstroke.	1,166 (85.99)
K9. Insufficient fluid intake may induce heatstroke.	1,267 (93.44)
K10. Marathons, large sports events, and large-scale military exercises and training are scenarios where heatstroke is more likely to occur.	1,290 (95.13)
K11. Once heatstroke occurs, even in extremely hot environments, the patient should not be moved.	963 (71.02)
K12. On-site treatment for heatstroke patients should involve laying them flat, tilting their head to one side, unfastening buttons and belts, and removing outer clothing to facilitate breathing and heat dissipation.	1,331 (98.16)
K13: On-site treatment for heatstroke includes applying ice, continuous fanning, spraying cold water, and wrapping with ice blankets.	1,188 (87.61)
K15. If heatstroke-induced coma occurs during physical training, and the person quickly regains consciousness, hospital treatment may not be necessary.	1,020 (75.22)
K16. Traditional Chinese medicines such as mint and patchouli can have a good therapeutic effect on heatstroke to some extent.	1,253 (92.40)

The responses to the attitude items indicated that most of the participants have a very positive or positive attitude toward all the items, specifically: 64.97 % strongly agree that knowledge about heatstroke is important for prevention and treatment (A1). Moreover, 65.56% strongly approved that employers should provide more measures to protect employees from heatstroke (A4). In addition, 51.62% were very worried about themselves or their relative experiencing heatstroke in the high temperatures (A5), and 59.14% were very willing to assist in the treatment of patients (A8) (Table 3).

**Table 3:** Attitudes.

	**N (%)**				
	**Strongly agree**	**Agree**	**Neutral**	**Disagree**	**Strongly disagree**
A1. Acquiring knowledge related to heatstroke is crucial for the effective prevention and treatment of heatstroke among the general public.	881 (64.97)	450 (33.19)	24 (1.77)	1 (0.07)	/
A2. I am willing to share knowledge about the prevention and treatment of heatstroke with my family, friends, or colleagues.	814 (60.03)	518 (38.2)	24 (1.77)	/	/
A3. Health authorities should strengthen public education and promotion regarding heatstroke.	835 (61.58)	496 (36.58)	25 (1.84)	/	/
A4. In high-temperature working environments, employers should provide more measures to protect employees from heatstroke.	889 (65.56)	449 (33.11)	16 (1.18)	2 (0.15)	/
A5. I am concerned about the possibility of myself or my relative experiencing heatstroke in high temperatures.	700 (51.62)	568 (41.89)	78 (5.75)	9 (0.66)	1 (0.07)
A6. I acknowledge the importance of on-site first aid measures in the treatment of heatstroke.	853 (62.91)	476 (35.1)	25 (1.84)	2 (0.15)	/
A7. I recognize the importance of “prevention is better than cure” for heatstroke, such as using air conditioning, avoiding prolonged exposure to unfavorable environments, and assessing physical fitness before engaging in sports or labor.	816 (60.18)	496 (36.58)	33 (2.43)	10 (0.74)	1 (0.07)
A8. If I were to encounter someone with heatstroke, I would be willing to actively assist in their treatment.	802 (59.14)	520 (38.35)	32 (2.36)	2 (0.15)	/

Moreover, 49.19% of them were very willing to participate in training related to heatstroke first aid (P2). In hot weather, 59% were well hydrated (P5), 56.49% were concerned about the possible effects of medication on thermoregulation (P6), 58.78% were alerted to symptoms such as burning skin and dizziness (P7), and 58.33% prompted those around them to take precautions (P8) (Table 4).

**Table 4:** Practices.

	**N (%)**				
	**Very consistent**	**Quite consistent**	**Consistent**	**Quite inconsistent**	**Very inconsistent**
P1. In my daily life, I acquire knowledge about heatstroke through various channels such as short videos, public accounts, and informative articles.	676 (49.85)	436 (32.15)	211 (15.56)	25 (1.84)	8 (0.59)
P2. I participate in training related to on-site first aid for heatstroke.	574 (42.33)	385 (28.39)	323 (23.82)	57 (4.2)	17 (1.25)
P3. Before engaging in physical labor or high-intensity physical training, I assess my own health condition.	667 (49.19)	441 (32.52)	213 (15.71)	28 (2.06)	7 (0.52)
P4. During the summer or in hot weather, I prepare items like a thermometer, blood pressure monitor, ice packs, and cold towels at home to handle the occurrence of heatstroke.	686 (50.59)	378 (27.88)	230 (16.96)	52 (3.83)	10 (0.74)
P5. In hot weather, I ensure to replenish an adequate amount of fluids to maintain internal water and electrolyte balance.	800 (59)	408 (30.09)	133 (9.81)	13 (0.96)	2 (0.15)
P6. In hot weather, I carefully read the medication instructions before taking any drugs, paying attention to their potential impact on temperature regulation.	766 (56.49)	385 (28.39)	177 (13.05)	23 (1.7)	5 (0.37)
P7. Symptoms such as skin burning and dizziness in hot weather immediately capture my attention.	797 (58.78)	412 (30.38)	139 (10.25)	7 (0.52)	1 (0.07)
P8. In hot weather, when outdoors with family or friends, I advise those around me to be mindful of preventing heatstroke or other central nervous system-related conditions.	791 (58.33)	405 (29.87)	147 (10.84)	11 (0.81)	2 (0.15)

Pearson's analysis was performed to assess the relationship between knowledge, attitude, and practice. It demonstrated that knowledge and attitudes were positively correlated (*r* = 0.177, *P* < 0.001), and knowledge and practices were also positively correlated (*r* = 0.079, *P* = 0.004). Additionally, there was a positive correlation between attitudes and practices (*r* = 0.454, *P* < 0.001) (Supplementary Table S1).

Multivariate analysis showed that occupation of outdoor worker (O*R* = 0.476, 95% CI: 0.259–0.876, *P* = 0.017) was independently associated with sufficient knowledge (Supplementary Table S2). Meanwhile, knowledge (O*R* = 1.208, 95% CI: 1.108–1.318, *P* < 0.001), age (O*R* = 0.982, 95% CI: 0.968–0.996, *P* = 0.013), master's degree and above (O*R* = 3.929, 95% CI: 1.108–13.935, *P* = 0.034), and monthly per capita income (CNY) of 10,000–20,000 CNY (O*R* = 2.075, 95% CI: 1.108–3.884, *P* = 0.023) were independently associated with active attitudes (Supplementary Table S3). Furthermore, attitudes (O*R* = 1.287, 95% CI: 1.225–1.351, *P* < 0.001), monthly per capita income (CNY) of 2,000–5,000 CNY (O*R* = 2.180, 95% CI: 1.187–4.006, *P* = 0.012), having regular exercise (O*R* = 1.829, 95% CI: 1.335–2.505, *P* < 0.001), having medical check-ups every 6 months or less (O*R* = 1.370, 95% CI: 1.026–1.830, *P* = 0.033), and heatstroke having been experienced by family members or friends (O*R* = 2.267, 95% CI: 1.203–4.273, *P* = 0.011) were independently associated with proactive practice (Supplementary Table S4).

**Figure 1:**
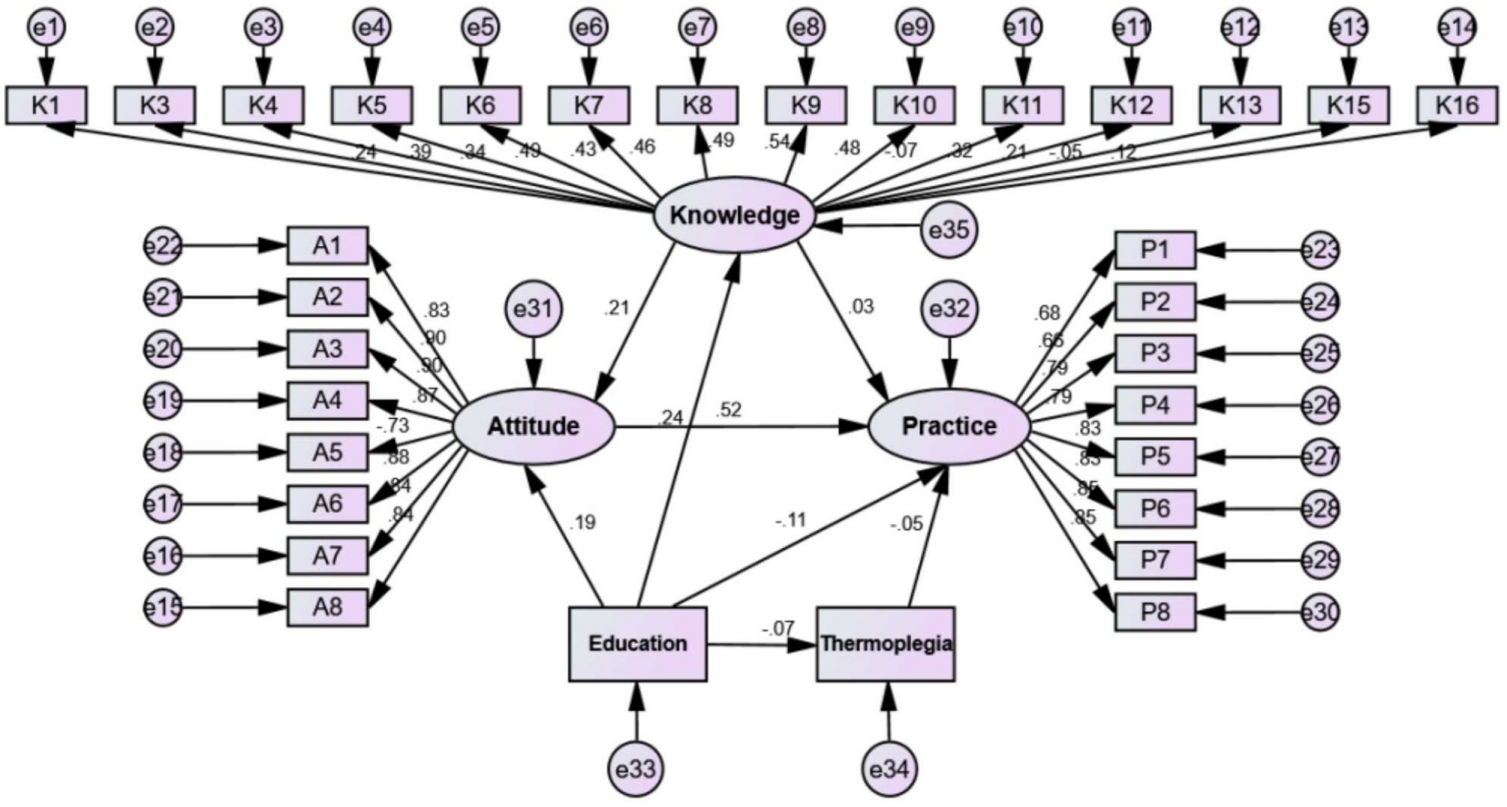
Structural equation modeling.

The fitting index of the structural equation model (CMIN/DF = 5.180; RMSEA = 0.056; IFI = 0.908; TLI = 0.900; CFI = 0.908) outperformed the respective threshold value, signifying that the data satisfactorily fit the structural model (Supplementary Table S5). The results showed that education had direct effects on knowledge (β = 0.017, *p* < 0.001), attitudes (β = 0.123, *p* < 0.001), and practices (β = −0.094, *p* < 0.001). Moreover, knowledge had direct effects on attitudes (β = 1.920, *p* < 0.001), and attitudes had direct effects on practices (β = 0.642, *p* < 0.001) (Supplementary Table S6 and Figure 1).

## Discussion

This study revealed that the general community population have sufficient knowledge, active attitudes and proactive practices toward the heatstroke.

Participants demonstrated commendable awareness regarding common symptoms and risk factors for heatstroke, such as high temperatures and humidity. However, notable knowledge gaps exist concerning the appropriate response to heatstroke-induced coma during physical training and the potential therapeutic effects of traditional Chinese medicines. Previous studies have highlighted the importance of public knowledge in preventing heat-related illnesses (18, 19), yet disparities in specific knowledge areas underscore the need for targeted educational campaigns.

Most of the participants exhibited positive inclinations toward acquiring knowledge and sharing it with others. However, concerns arise regarding the perceived necessity of public education on heatstroke and employer responsibilities in high-temperature work environments. These findings align with research emphasizing the role of positive attitudes in promoting health-related behaviors (20, 21), yet the identified gaps suggest the potential for reinforcing educational initiatives, especially in workplace settings. Recognizing the positive attitudes toward employer responsibilities in high-temperature work environments, workplace interventions are crucial.

In terms of practices, participants reported consistent engagement in acquiring knowledge through various channels and assessing their health before physical exertion. However, disparities exist, with fewer individuals engaging in on-site first aid training and preparing home items for heatstroke prevention. These findings echo the importance of knowledge translation into practical behaviors (22, 23) while highlighting areas for intervention, particularly in enhancing first aid preparedness. Collaboration with local healthcare providers and community organizations can facilitate the implementation of such programs (24). While many participants reported consistent practices in acquiring knowledge and assessing health before physical exertion, there is room for improvement in home-based preventive measures. Public health campaigns should encourage individuals to prepare home items, such as thermometers, blood pressure monitors, and ice packs, to handle potential heatstroke occurrences. Practical guidance on assembling a heatstroke preparedness kit can be disseminated through various channels, including social media and community workshops (25).

Notably, urban residents demonstrated higher mean scores in both knowledge and attitude compared to their rural counterparts in this study, underscoring the potential influence of urbanization on awareness and attitudes toward heatstroke. This aligns with existing literature suggesting that urban areas often have better access to health information and resources, potentially contributing to heightened knowledge and more positive attitudes (26, 27). This finding resonates with numerous studies emphasizing the positive correlation between education and health-related knowledge (28, 29). The pronounced impact of occupation, particularly the lower knowledge scores among outdoor workers, aligns with previous research indicating occupational disparities in health knowledge (30, 31). Additionally, the positive correlation between monthly per capita income and knowledge and attitude scores emphasizes the role of socio-economic factors in shaping KAP outcomes, reflecting findings from studies on health literacy and socio-economic status (32, 33). This study highlights the significant influence of urbanization, occupation, and socio-economic status on the KAP toward heatstroke, revealing key disparities and potential areas for targeted educational and resource allocation efforts.

Moreover, the results of SEM demonstrated that education exerted direct positive effects on knowledge, attitudes, and practices, aligning with the notion that educational interventions can be instrumental in fostering a holistic approach to heatstroke prevention (34), suggesting that educational interventions could be pivotal in enhancing heatstroke prevention measures. However, the negative association between education and practices might indicate that while education improves knowledge and attitudes, it does not always translate into practical application. This paradox warrants further investigation, possibly indicating a gap between theoretical understanding and real-world implementation that needs to be addressed through more practical, hands-on educational strategies.

The limitations of the study include its cross-sectional nature, which precludes causal inferences, and the potential for self-selection bias. Additionally, the web-based distribution of the questionnaire may have excluded individuals with limited internet access or technological proficiency. Future research should aim to include these populations to provide a more comprehensive understanding of the community's KAP toward heatstroke.

Nevertheless, this study still holds significant clinical relevance as it offers valuable insights into the current state of knowledge, attitudes, and practices toward heatstroke, which can guide the development of targeted educational and preventive strategies in healthcare settings.

## Conclusion

The findings revealed that the general community population have sufficient knowledge, active attitudes and proactive practices toward the heatstroke. However, there is still room for improvement and it is necessary to develop and implement educational initiatives and interventions designed to further enhance their KAP toward heatstroke. Employers should implement comprehensive measures to protect outdoor workers, providing adequate rest time, hydration facilities, and heat-resistant clothing. Educational programs within workplaces can further enhance employees' awareness of heatstroke risks and preventive measures.

## Data availability statement

The original contributions presented in the study are included in the article/Supplementary material, further inquiries can be directed to the corresponding author.

## Ethics statement

The studies involving humans were approved by the Ethics Committee of Dongyang People's Hospital (Approval No. 2023-YX-289). The studies were conducted in accordance with the local legislation and institutional requirements. The participants provided their written informed consent to participate in this study.

## Author contributions

YX: Conceptualization, Data curation, Investigation, Writing – original draft, Writing – review & editing. JC: Conceptualization, Data curation, Formal analysis, Writing – review & editing. JD: Conceptualization, Data curation, Formal analysis, Writing – review & editing. YJ: Conceptualization, Data curation, Investigation, Writing – original draft, Writing – review & editing.
