# Supplementary material for: Knowledge, attitudes, and practices among the general community population toward heatstroke
Source: Front Public Health. 2024 May 24;12:1373025. doi: 10.3389/fpubh.2024.1373025 (PMC11157029; doi:10.3389/fpubh.2024.1373025)
Supplement: Supplementary file 1 [file Table_1.DOC]

**Table S1** Pearson’s analysis

|  | Knowledge | Attitude | Practice |
| --- | --- | --- | --- |
| Knowledge | 1 |  |  |
| Attitude | 0.177 (P<0.001) | 1 |  |
| Practice | 0.079 (P=0.004) | 0.454 (P<0.001) | 1 |

**Table S2** Multivariate analysis of knowledge

|  | Univariate logistic regression | | Multivariate logistic regression | |
| --- | --- | --- | --- | --- |
|  | OR (95%CI) | P | OR (95%CI) | P |
| **Gender** |  |  |  |  |
| Male | 1.085 (0.679-1.735) | 0.733 |  |  |
| Female | ref |  |  |  |
| **Age (years)** | 0.962 (0.942-0.982) | <0.001 | 0.989 (0.964-1.015) | 0.397 |
| **BMI (kg/m^2^)** |  |  |  |  |
| <18.5 | 0.720 (0.213-2.435) | 0.597 |  |  |
| 18.5-23.9 | 0.935 (0.328-2.665) | 0.900 |  |  |
| 24-27.9 | 1.242 (0.402-3.840) | 0.707 |  |  |
| ≥28 | ref |  |  |  |
| **Residence** |  |  |  |  |
| Rural | ref |  |  |  |
| Urban | 1.357 (0.869-2.118) | 0.179 |  |  |
| **Marital Status** |  |  |  |  |
| Unmarried | ref |  | ref |  |
| Married | 0.297 (0.092-0.953) | 0.041 | 0.444 (0.127-1.545) | 0.202 |
| Other | 0.287 (0.056-1.474) | 0.135 | 0.478 (0.086-2.662) | 0.400 |
| **Education** |  |  |  |  |
| Junior high school and below | ref |  | ref |  |
| High school/Technical school | 1.124 (0.586-2.155) | 0.725 | 0.941 (0.475-1.867) | 0.863 |
| College/Bachelor's degree | 3.275 (1.796-5.969) | <0.001 | 1.974 (0.968-4.026) | 0.062 |
| Master's degree and above | 4.917 (0.632-38.257) | 0.128 | 3.251 (0.393-26.881) | 0.274 |
| **Occupation** |  |  |  |  |
| Outdoor worker (e.g., sanitation worker, traffic police, construction worker, etc.) | 0.343 (0.194-0.606) | <0.001 | 0.476 (0.259-0.876) | 0.017 |
| Indoor worker (clerk, accountant, designer, etc.) | ref |  | ref |  |
| Occupation involving both indoor and outdoor activities | 0.884 (0.393-1.985) | 0.765 | 1.119 (0.485-2.586) | 0.792 |
| Unemployed, retired, or other non-working situations | 0.314 (0.176-0.559) | <0.001 | 0.539 (0.273-1.063) | 0.074 |
| **Monthly per capita income (CNY)** |  |  |  |  |
| <2,000 | ref |  | ref |  |
| 2,000-5,000 | 3.199 (1.459-7.014) | 0.004 | 2.045 (0.887-4.711) | 0.093 |
| 5,000-10,000 | 3.307 (1.567-6.979) | 0.002 | 1.787 (0.774-4.122) | 0.174 |
| 10,000-20,000 | 3.077 (1.330-7.121) | 0.009 | 1.558 (0.609-3.986) | 0.354 |
| >20,000 | 2.273 (0.915-5.649) | 0.077 | 1.224 (0.452-3.313) | 0.690 |
| **Medical Insurance** |  |  |  |  |
| Uninsured | ref |  |  |  |
| Social insurance only | 1.693 (0.688-4.164) | 0.252 |  |  |
| Comprehensive coverage | 1.915 (0.770-4.759) | 0.162 |  |  |
| **Smoking or Drinking** |  |  |  |  |
| Smoking only | 1.242 (0.440-3.506) | 0.682 |  |  |
| Drinking only | 0.640 (0.318-1.288) | 0.211 |  |  |
| Both | 0.856 (0.414-1.767) | 0.674 |  |  |
| Neither | ref |  |  |  |
| **Regular Exercise** |  |  |  |  |
| Yes | 0.866 (0.550-1.364) | 0.535 |  |  |
| No | ref |  |  |  |
| **Frequency of Medical Check-ups** |  |  |  |  |
| Every six months or less | 1.113 (0.711-1.742) | 0.640 |  |  |
| Once a year | ref |  |  |  |
| **Average nightly sleep duration in the past week** |  |  |  |  |
| 5 hours or less | ref |  | ref |  |
| 5 to 7 hours (including 7 hours) | 2.123 (1.065-4.232) | 0.032 | 1.303 (0.622-2.730) | 0.483 |
| 7 hours or more | 1.707 (0.806-3.615) | 0.162 | 1.006 (0.442-2.291) | 0.989 |
| **Has there been any incidence of heatstroke?** |  |  |  |  |
| Personal experience of heatstroke has occurred. | 1.080 (0.329-3.549) | 0.899 |  |  |
| Heatstroke has been experienced by family members or friends. | 1.539 (0.610-3.884) | 0.361 |  |  |
| There have been no recent reports of heatstroke in the surrounding area. | ref |  |  |  |

**Table S3 Multivariate analysis of attitudes**

|  | Univariate logistic regression | | Multivariate logistic regression | |
| --- | --- | --- | --- | --- |
|  | OR (95%CI) | P | OR (95%CI) | P |
| **Knowledge** | 1.270 (1.167-1.381) | <0.001 | 1.208 (1.108-1.318) | <0.001 |
| **Gender** |  |  |  |  |
| Male | 0.846 (0.654-1.093) | 0.201 |  |  |
| Female | ref |  |  |  |
| **Age (years)** | 0.973 (0.961-0.985) | <0.001 | 0.982 (0.968-0.996) | 0.013 |
| **BMI (kg/m^2^)** |  |  |  |  |
| <18.5 | 1.112 (0.550-2.248) | 0.768 |  |  |
| 18.5-23.9 | 1.061 (0.599-1.881) | 0.839 |  |  |
| 24-27.9 | 1.076 (0.587-1.972) | 0.813 |  |  |
| ≥28 | ref |  |  |  |
| **Residence** |  |  |  |  |
| Rural | ref |  |  |  |
| Urban | 1.085 (0.843-1.397) | 0.526 |  |  |
| **Marital Status** |  |  |  |  |
| Unmarried | ref |  |  |  |
| Married | 0.841 (0.553-1.278) | 0.418 |  |  |
| Other | 0.578 (0.273-1.221) | 0.151 |  |  |
| **Education** |  |  |  |  |
| Junior high school and below | ref |  | ref |  |
| High school/Technical school | 1.046 (0.672-1.629) | 0.841 | 0.891 (0.560-1.420) | 0.628 |
| College/Bachelor's degree | 2.020 (1.376-2.965) | <0.001 | 1.314 (0.840-2.056) | 0.232 |
| Master's degree and above | 5.867 (1.710-20.126) | 0.005 | 3.929 (1.108-13.935) | 0.034 |
| **Occupation** |  |  |  |  |
| Outdoor worker (e.g., sanitation worker, traffic police, construction worker, etc.) | 0.629 (0.453-0.874) | 0.006 | 0.854 (0.600-1.215) | 0.379 |
| Indoor worker (clerk, accountant, designer, etc.) | ref |  | ref |  |
| Occupation involving both indoor and outdoor activities | 0.937 (0.635-1.381) | 0.741 | 1.178 (0.784-1.769) | 0.430 |
| Unemployed, retired, or other non-working situations | 0.599 (0.426-0.842) | 0.003 | 1.058 (0.709-1.579) | 0.783 |
| **Monthly per capita income (CNY)** |  |  |  |  |
| <2,000 | ref |  | ref |  |
| 2,000-5,000 | 1.964 (1.159-3.327) | 0.012 | 1.496 (0.848-2.639) | 0.164 |
| 5,000-10,000 | 2.254 (1.348-3.768) | 0.002 | 1.594 (0.902-2.817) | 0.109 |
| 10,000-20,000 | 2.891 (1.638-5.104) | <0.001 | 2.075 (1.108-3.884) | 0.023 |
| >20,000 | 2.394 (1.283-4.466) | 0.006 | 1.610 (0.821-3.158) | 0.166 |
| **Medical Insurance** |  |  |  |  |
| Uninsured | ref |  |  |  |
| Social insurance only | 1.557 (0.883-2.745) | 0.126 |  |  |
| Comprehensive coverage | 1.752 (0.990-3.099) | 0.054 |  |  |
| **Smoking or Drinking** |  |  |  |  |
| Smoking only | 0.982 (0.583-1.656) | 0.947 |  |  |
| Drinking only | 0.812 (0.524-1.258) | 0.351 |  |  |
| Both | 0.827 (0.550-1.245) | 0.363 |  |  |
| Neither | ref |  |  |  |
| **Regular Exercise** |  |  |  |  |
| Yes | 1.000 (0.772-1.294) | 0.998 |  |  |
| No | ref |  |  |  |
| **Frequency of Medical Check-ups** |  |  |  |  |
| Every six months or less | 1.137 (0.884-1.462) | 0.318 |  |  |
| Once a year | ref |  |  |  |
| **Average nightly sleep duration in the past week** |  |  |  |  |
| 5 hours or less | ref |  | ref |  |
| 5 to 7 hours (including 7 hours) | 1.570 (1.008-2.446) | 0.046 | 1.133 (0.704-1.823) | 0.606 |
| 7 hours or more | 1.517 (0.939-2.451) | 0.089 | 1.062 (0.632-1.785) | 0.821 |
| **Has there been any incidence of heatstroke?** |  |  |  |  |
| Personal experience of heatstroke has occurred. | 1.364 (0.675-2.756) | 0.388 |  |  |
| Heatstroke has been experienced by family members or friends. | 1.466 (0.906-2.373) | 0.119 |  |  |
| There have been no recent reports of heatstroke in the surrounding area. | ref |  |  |  |

**Table S4 Multivariate analysis of practices**

|  | Univariate logistic regression | | Multivariate logistic regression | |
| --- | --- | --- | --- | --- |
|  | OR (95%CI) | P | OR (95%CI) | P |
| **Knowledge** | 1.140 (1.046-1.242) | 0.003 | 1.021 (0.928-1.124) | 0.672 |
| **Attitudes** | 1.290 (1.232-1.352) | <0.001 | 1.287 (1.225-1.351) | <0.001 |
| **Gender** |  |  |  |  |
| Male | 0.841 (0.642-1.102) | 0.210 |  |  |
| Female | ref |  |  |  |
| **Age (years)** | 0.999 (0.986-1.013) | 0.937 |  |  |
| **BMI (kg/m^2^)** |  |  |  |  |
| <18.5 | 1.086 (0.475-2.484) | 0.845 |  |  |
| 18.5-23.9 | 0.735 (0.377-1.433) | 0.366 |  |  |
| 24-27.9 | 0.665 (0.332-1.334) | 0.251 |  |  |
| ≥28 | ref |  |  |  |
| **Residence** |  |  |  |  |
| Rural | ref |  |  |  |
| Urban | 1.278 (0.980-1.665) | 0.070 |  |  |
| **Marital Status** |  |  |  |  |
| Unmarried | ref |  |  |  |
| Married | 1.467 (0.990-2.176) | 0.056 |  |  |
| Other | 1.093 (0.504-2.373) | 0.821 |  |  |
| **Education** |  |  |  |  |
| Junior high school and below | ref |  |  |  |
| High school/Technical school | 1.090 (0.666-1.783) | 0.732 |  |  |
| College/Bachelor's degree | 1.357 (0.893-2.065) | 0.153 |  |  |
| Master's degree and above | 0.850 (0.372-1.941) | 0.700 |  |  |
| **Occupation** |  |  |  |  |
| Outdoor worker (e.g., sanitation worker, traffic police, construction worker, etc.) | 1.226 (0.847-1.776) | 0.280 |  |  |
| Indoor worker (clerk, accountant, designer, etc.) | ref |  |  |  |
| Occupation involving both indoor and outdoor activities | 1.308 (0.863-1.984) | 0.206 |  |  |
| Unemployed, retired, or other non-working situations | 0.835 (0.585-1.192) | 0.321 |  |  |
| **Monthly per capita income (CNY)** |  |  |  |  |
| <2,000 | ref |  | ref |  |
| 2,000-5,000 | 2.702 (1.556-4.694) | <0.001 | 2.180 (1.187-4.006) | 0.012 |
| 5,000-10,000 | 2.197 (1.298-3.717) | 0.003 | 1.518 (0.842-2.735) | 0.165 |
| 10,000-20,000 | 2.419 (1.360-4.306) | 0.003 | 1.407 (0.739-2.680) | 0.299 |
| >20,000 | 1.842 (0.986-3.441) | 0.056 | 1.054 (0.519-2.140) | 0.884 |
| **Medical Insurance** |  |  |  |  |
| Uninsured | ref |  | ref |  |
| Social insurance only | 1.509 (0.842-2.703) | 0.167 | 1.107 (0.577-2.126) | 0.760 |
| Comprehensive coverage | 1.997 (1.107-3.604) | 0.022 | 1.366 (0.698-2.673) | 0.363 |
| **Smoking or Drinking** |  |  |  |  |
| Smoking only | 0.794 (0.470-1.342) | 0.390 |  |  |
| Drinking only | 0.719 (0.459-1.126) | 0.150 |  |  |
| Both | 0.890 (0.574-1.381) | 0.603 |  |  |
| Neither | ref |  |  |  |
| **Regular Exercise** |  |  |  |  |
| Yes | 1.779 (1.330-2.381) | <0.001 | 1.829 (1.335-2.505) | <0.001 |
| No | ref |  | ref |  |
| **Frequency of Medical Check-ups** |  |  |  |  |
| Every six months or less | 1.594 (1.225-2.075) | 0.001 | 1.370 (1.026-1.830) | 0.033 |
| Once a year | ref |  | ref |  |
| **Average nightly sleep duration in the past week** |  |  |  |  |
| 5 hours or less | ref |  |  |  |
| 5 to 7 hours (including 7 hours) | 1.297 (0.807-2.082) | 0.282 |  |  |
| 7 hours or more | 1.432 (0.855-2.399) | 0.173 |  |  |
| **Has there been any incidence of heatstroke?** |  |  |  |  |
| Personal experience of heatstroke has occurred. | 2.148 (0.906-5.089) | 0.083 | 1.939 (0.787-4.775) | 0.150 |
| Heatstroke has been experienced by family members or friends. | 2.553 (1.384-4.709) | 0.003 | 2.267 (1.203-4.273) | 0.011 |
| There have been no recent reports of heatstroke in the surrounding area. | ref |  | ref |  |

**Table S5** Model fitness indices for the KAP structural equation model

| **Goodness-of-Fit Indices** | **Ideal standards** | **Measured results** |
| --- | --- | --- |
| **CMIN/DF** | 1-3 excellent，3-5 good | 5.180 |
| **RMSEA** | <0.08 good | 0.056 |
| **IFI** | >0.8 good | 0.908 |
| **TLI** | >0.8 good | 0.900 |
| **CFI** | >0.8 good | 0.908 |

**Table S6** Test results of the hypothesis

| Hypothesized paths | | | Estimate | S.E. | C.R. | P |
| --- | --- | --- | --- | --- | --- | --- |
| Knowledge | <--- | Education | 0.017 | 0.003 | 5.396 | <0.001 |
| Attitude | <--- | Knowledge | 1.920 | 0.394 | 4.876 | <0.001 |
| Attitude | <--- | Education | 0.123 | 0.019 | 6.620 | <0.001 |
| Experience of heatstroke | <--- | Education | -0.045 | 0.018 | -2.487 | 0.013 |

| Practice | <--- | Attitude | 0.642 | 0.039 | 16.336 | <0.001 |
| --- | --- | --- | --- | --- | --- | --- |
| Practice | <--- | Knowledge | 0.367 | 0.359 | 1.023 | 0.306 |
| Practice | <--- | Education | -0.094 | 0.021 | -4.385 | <0.001 |
| Practice | <--- | Experience of heatstroke | -0.067 | 0.030 | -2.187 | 0.029 |
